# Toxic effect of three imidazole ionic liquids on two terrestrial plants

**DOI:** 10.1515/biol-2020-0051

**Published:** 2020-07-06

**Authors:** Lin Ma, Qirong Lin, Yuhua Song, Bo Zhao, Mingjin Fan

**Affiliations:** Institution Shaanxi Key Laboratory of Phytochemistry, College of Chemistry & Chemical Engineering, Baoji University of Arts and Sciences, Baoji 721013, China

**Keywords:** imidazole ionic liquids, terrestrial plant, toxic effect, cell membrane permeability

## Abstract

To determine the toxic effect of three imidazole ionic liquids (IILs) in terrestrial monocotyledonous and dicotyledonous plants, three IILs (1-butyl-3-methylimidazole tetrafluoroborate, 1-butyl-3-methylimidazole hexafluorophosphate, and butyl-3-methylimidazolium bi-[(trifluoromethyl)sulfonyl]imide) were investigated using rice and capsicum as target toxicity models. In hydroponic experiments, increasing the concentration of the IILs led to a decrease in the seed germination rate, a decrease in the reduced stem and root lengths, and an increase in the inhibition rate of the stem and root lengths; in addition, as the concentration increased, the reducing sugar content of rice and capsicum seedling leaves and roots first increased and then decreased, while permeability of the cell membranes of the stems and roots of the two plants also gradually increased. In terms of the effects on these indices in rice, the ranking of these three IIL anions was [TF2N]- > [PF6]- > [BF4]-; in terms of the effects on capsicum, the sequence was [BF4]- > [TF2N]- > [PF6]-. These findings provide a theoretical reference for the next step in the synthesis and the use of green ionic liquids.

## 1 Introduction

The application of ionic liquids (ILs) in the field of tribology has attracted the attention of many researchers around the world since Liu Weimin’s research group first reported in 2001 that ILs are a group of multipurpose lubricants with excellent performance [1]. ILs are liquid salts composed of larger cations and smaller anions with a melting point below 100°C [2,3,4,5]. As a lubricant and lubricating additive, they have advantages of lower vapor pressure, no volatility, recyclability, high chemical and thermal stability, and strong solubility. Therefore, ILs are considered to be high-performance lubricants or additives used in special fields such as the aerospace and computer industry [6,7,8]. In addition to applications in lubrication, they are also used in biocatalysis, solvents, herbicides, and detergents [9,10,11,12].

Following their extensive use, some ILs will inevitably enter the local environment [13,14]. Because of their high stability, they may be a source of persistent pollution in the environment [15]. Current studies [16] have shown that the presence of ILs in the environment may affect organisms at all levels of the food chain and eventually threaten human health [17]. Currently, there is some research on the toxicity of ionic liquids to organisms. Furthermore, previous studies have shown that different anionic factors have different toxic effects [18,19,20]. Therefore, before the large-scale application of ILs, it is necessary to study their toxic effects on organisms and the related mechanisms [21].

In this study, two plants (rice and capsicum) were selected as the toxicity models for three imidazole ionic liquids (IILs). Rice is a typical monocotyledonous plant. As one of the main cereals, it is consumed by half of the world’s population [22] and has been recommended by the World Organization of Economic Cooperation and Development as the research species for toxicology [23]. Capsicum is a typical dicotyledonous plant, and it is one of the most widely consumed condiments in the world [24]. Both of them are characterized by simple planting, fast growth, and strong sensitivity. Many researchers have used these two plants for related toxicity experiments [25,26].

To investigate the toxic effects of IIL anions, we selected three IILs (1-butyl-3-methylimidazole tetrafluoroborate (LB_104_), 1-butyl-3-methylimidazole hexafluorophosphate (LP_104_), butyl-3-methylimidazolium bis[(trifluoromethyl)sulfonyl]imide (LF_104_)), and rice and capsicum as toxicity models. The 50% effective concentration (EC_50_) of the three IILs on germination rate, root length, stem length, and other growth indicators was used to determine the toxic effect of the three anions on the two model plants. Furthermore, the possible toxicity of three IILs to the cell membrane of the plants was studied by the electrolyte content and soluble reducing sugar content in the rhizome cells. This study provides a theoretical reference for the next step in the synthesis and the use of green ionic liquids.

## 2 Materials and methods

### 2.1 Materials

Acetone (≥99.5%) was purchased from Shanghai TITAN Technology Co., Ltd, and reducing sugar content kit from Beijing Solarbio Science & Technology Co., Ltd. LB_104_, LP_104_, and LF_104_ (Table 1) were all purchased from the Green Chemistry Research and Development Center of the Lanzhou Institute of Chemical Physics, Chinese Academy of Sciences (purity > 98%). The variety of rice used was Hangzhou Liangyouxiangzhan purchased from Hubei Fuyue Agricultural Group Co., Ltd; the variety of capsicum used was GaoKe Changshun Prince purchased from Yangling Agricultural High-tech Development Co., Ltd. The important equipment included centrifuge (H/t16mm, Hunan Hexi instrument & Equipment Co., Ltd), constant temperature oscillation incubator (Ts-1102 *, Shanghai Tiancheng Experimental Instrument Manufacturing Co., Ltd), conductivity meter (Shanghai Yulong Instrument Co., Ltd), and enzyme labeling instrument (Gen Co., Ltd).

**Table 1:** Chemical structure and abbreviation of three imidazole ionic liquids

Ionic liquids	Abbreviations	Chemical structure
1-Butyl-3-methylimidazolium tetrafluoroborate	LB_104_	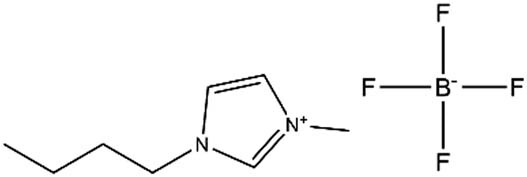
1-Butyl-3-methylimidazolium hexafluorophosphate	LP_104_	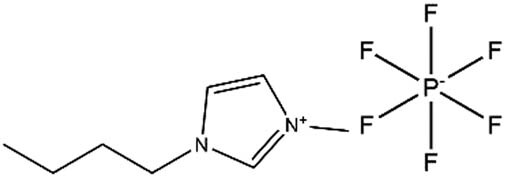
Butyl-3-methylimidazolium bis[(trifluoromethyl)sulfonyl]imide	LF_104_	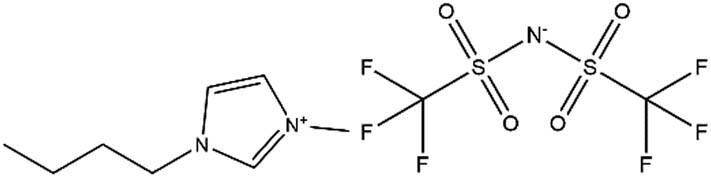

### 2.2 Methods

#### 2.2.1 Toxicity experiments of three IILs on two kinds of plants

The hydroponic experiments were conducted using the two selected plant species. First, the rice and capsicum seeds were placed in a 12-well plate with two layers of filter paper and a small amount of ionic liquid solution (the concentration of each IL solution was 0.000, 1.000, 2.500, 5.000, 7.500 and 10.000 g/L). The treated 12-well plate was placed in a 27°C incubator for 7-day germination and the seedling growth test. During the test process, an equal amount of de-ionized water was added daily to keep the seeds moist, with the light on for 14 h and off for 10 h every day. Based on the dissolution of the IL samples, different solvents were used as the blank control group; for LB_104_, deionized water was the control group; for LP_104_ and LF_104_ ionic liquid, acetone was used as the control as LP_104_ and LF_104_ were insoluble in water.

Based on the International Rules for Seed Testing [27], the germination rate (GR) and the rooting rate (GP) were calculated on the 7th day. According to the study by Liu et al. [28], seeds with a germ of larger than 2 mm are considered to be germinated; meanwhile, the root and stem length of the seedlings were measured separately and compared with the control group. Based on these measurements, the root and stem inhibition rates of the IILs on the seedlings were estimated.

\text{GR}=\left(\text{Se}/\text{Sn}\right)\times 100 \%

where Se is all normal germinated seeds at the end of seed germination and Sn is the total number of seeds tested.

\text{GP}=\left({\text{Sn}}_{1}/{\text{Sn}}_{2}\right)\times 100 \%

where Sn_1_ is all normal rooting seeds at the end of seed germination and Sn_2_ is the total number of seeds tested.

\text{HP}={[}-({L}_{\text{p}}-{L}_{\text{c}})/{L}_{\text{c}}]\times 100 \%

where HP is the inhibition rate of the stem length, *L*_p_ is the stem length of the experiment group, and *L*_c_ is the stem length of the control group.

{H}_{\text{j}}={[}-({L}_{\text{j}}-{L}_{\text{d}})/{L}_{\text{d}}]\times 100 \%

where *H*_j_ is the inhibition rate of the root length, *L*_j_ is the root length of the experiment group, and *L*_d_ is the root length of the control group.

#### 2.2.2 The toxicity of three IILs to the cell membrane of rice and capsicum

The cell membrane has several important physiological functions. It keeps the intracellular environment stable but also selectively regulates the movement of substances in and out of the cell. Damage to the membrane will affect the flow of ions and the normal growth of cells. Ion leakage from the cells reflects damage to the cell membrane [29]. In this experiment, the effects of the three IILs on the roots or leaves of the two selected plants were measured in terms of electrical conductivity and reducing sugar content in the bath solution, reflecting damage to the cell membrane of the root cells. EC_50_ of the three IILs was then determined in rice and capsicum. The cultivating steps described in Section 2.2.1 were repeated. When studying the toxic mechanism of the three IILs on rice, the concentration of LB_104_ solution (dissolved in deionized water) was 0.050, 0.100, 0.500, 1.000, 1.500, 2.000, 2.500, and 3.000 g/L; the concentrations of the LF_104_ and LP_104_ solutions (soluble in acetone) were 0.050, 0.100, 0.250, 0.500, 0.750, and 1.000 g/L. When studying the toxic mechanism of the three IILs on capsicum, the concentrations of LB_104_ and LF_104_ solutions (dissolved in deionized water and acetone) were 0.100, 0.200, 0.400, 0.600, 0.800, and 1.000 g/L; the concentrations of LP_104_ ionic liquid solution (dissolved in acetone) were 0.200, 0.400, 0.800, 1.200, 1.600, and 2.000 g/L. Other conditions are the same as described in Section 2.2.1.

(1) Effects of three IILs on the content of inorganic salts in the cells of two sample plants
  This experiment made some modifications to the methods of Wu et al. [29]. The leaves or roots of the prepared plant seedlings were respectively intercepted for 0.5 g, cut into pieces, placed in centrifuge tubes with 10 mL deionized water, and then incubated at room temperature (25°C) for 1 h. The conductivity (*C*_1_) of the solution was measured separately. After heating the test sample at 80°C for 1 h, the conductivity (*C*_2_) was measured again. The amount of inorganic salt ion leakage is expressed as a percentage of the conductivity after heating.
  M( \% )=\left({C}_{1}/{C}_{2}\times \text{}100 \% \right)
  where *M* is the amount of inorganic salt ion leakage.
(2) Effects of three IILs on the content of intracellular reducing sugars of two sample plants
  The nitro salicylic acid method in the Solarbio reducing sugar content detection kit was used in this experiment: reagent 1 was added to 0.1 g of root or leaf and the material was mashed, and the supernatant was extracted by centrifuge at 3,000 r/s at 25°C for 20 min. Supernatant and reagent 2 (testing agent) were mixed in the centrifuge tube (EP) to be used as the testing tube, with water as a control tube. The detection agent was replaced with deionized water as a control tube; a standard tube of reducing sugar at a specific concentration is prepared, and the sample was replaced with deionized water to make a blank tube. Aliquots (200 µL) are taken into a 96-well plate, and the absorbance of standard tubes, control tubes, measuring tubes, and blank tubes was read at 540 nm.
  A standard curve was drawn according to the concentration and the absorbance of the standard tube (a standard tube − a blank tube), in which *x* is the absorbance and *y* is the concentration of standard tube (mg/mL). On this basis, the content of reducing sugar in the samples was also calculated, that is, substituting Δ*A* (a measuring/test tube − a control tube) into *x* to calculate *y*.
  N\hspace{.5em}\left(\text{µg}/\text{g}\right)=1,\hspace{-0.2em}000\hspace{.5em}\text{y}\hspace{.5em}V/W
  where *N* is the reducing sugar content in fresh weight, *V* is the volume of reagent 1, and *W* is the sample mass.

### 2.3 Data statistics and analysis

The data were analyzed and processed by Excel software, and the EC_50_ was estimated by nonlinear regression using GraphPad Prism software (GraphPad Software, Inc., La Jolla, CA, USA). The results were an average of the three measurements.

## 3 Results and discussion

### 3.1 Toxic effects of three IILs on two terrestrial plants

#### 3.1.1 Toxic effects of three IILs on rice

(1) Effects of three IILs on germination rate and rooting rate of rice seeds

**Figure 1:**
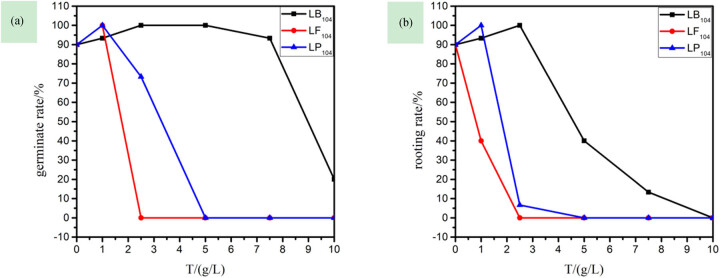
The relationship among germination rate (a), rooting rate (b), and the concentration of three kinds of ionic liquids in rice.

Seed germination is the beginning of the plant life cycle. At this stage, the IILs have already caused damage to the plants [30]. Figure 1 shows that the IILs have a great impact on the germination of rice seeds. With the increase of IIL concentration, the germination rate and the rooting rate of rice sharply decreased, especially at 2.50 and 5.00 g/L for LF_104_ and LP_104_, respectively, and both the germination rate and the rooting rate of the rice became 0, that is, no germination. In addition, the effects of the three anions on the root system of the plant were more obvious than on the stem, and in terms of the degree of sensitivity, their sequence was [TF_2_N]- > [PF_6_]- > [BF_4_]-.

(2) Effects of three IILs on stem and root length of rice seedlings

**Figure 2:**
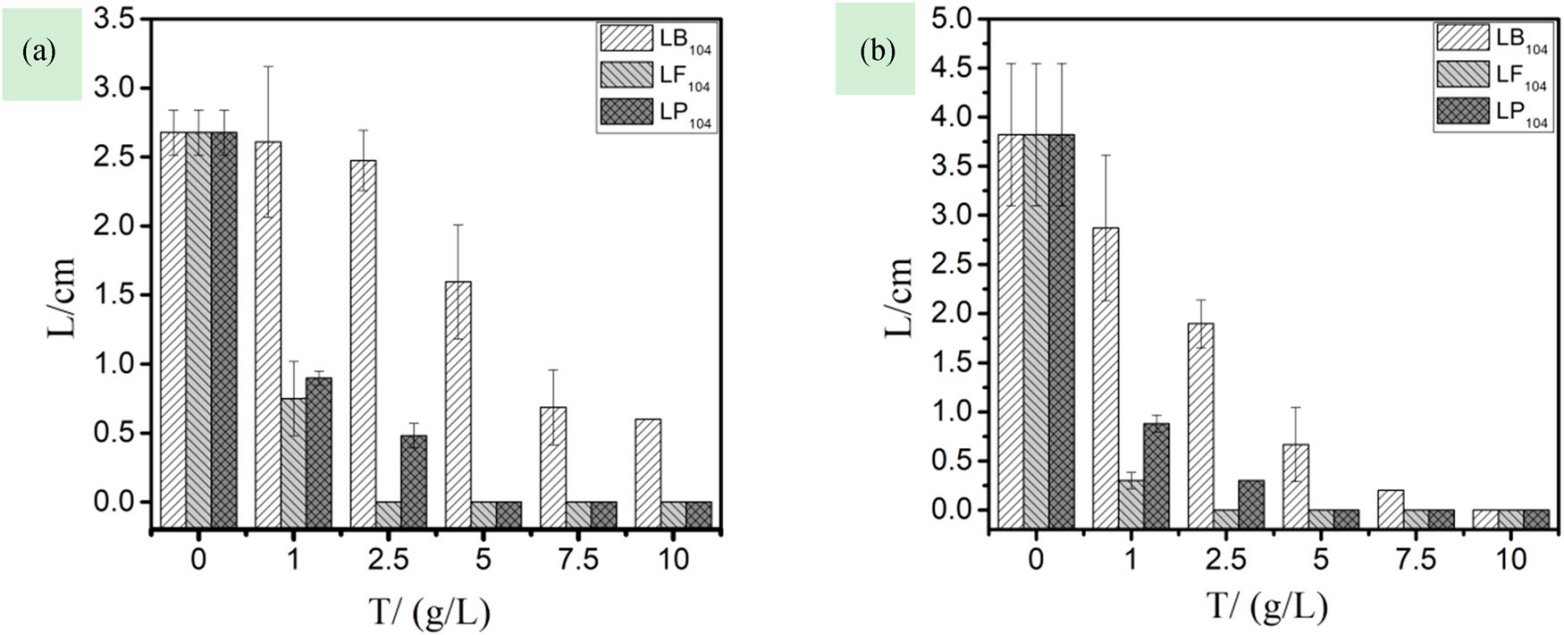
The relationship among stem length (a), root length (b), and the concentration of three kinds of ionic liquids in rice.

Stem length, root length, and dry weight are the most intuitive indicators of the plant growth. A decrease in any one of these parameters is an obvious indicator of the plant’s exposure to biotic or abiotic stress [31]. The stem and root lengths of a plant are the direct external manifestations of its growth. Figure 2 shows that the stem and root lengths of plants rapidly decreased with the increasing IL solution concentration, and the IL has a great effect on the root length of rice. At the same time, it was found that when the concentration of the ionic liquid was increased, the root system of rice became short, thick, and curved, and the number of lateral roots increased. It may be that the cells of the main root were easily damaged at the growth point. When cell division and proliferation were inhibited, the cells had accelerated the division speed near the growth point, leading to clustering of the lateral roots. Compared with the other two kinds of ionic liquids, LB_104_ had less effect on the two kinds of plants, while LP_104_ and LF_104_ have little difference, which may be related to their fluorine content. The order of sensitivity to the three anions is as follows: [TF_2_N]- > [PF_6_]- > [BF_4_]-.

(3) Effects of three IILs on the inhibition rate of rice stems and roots

**Figure 3:**
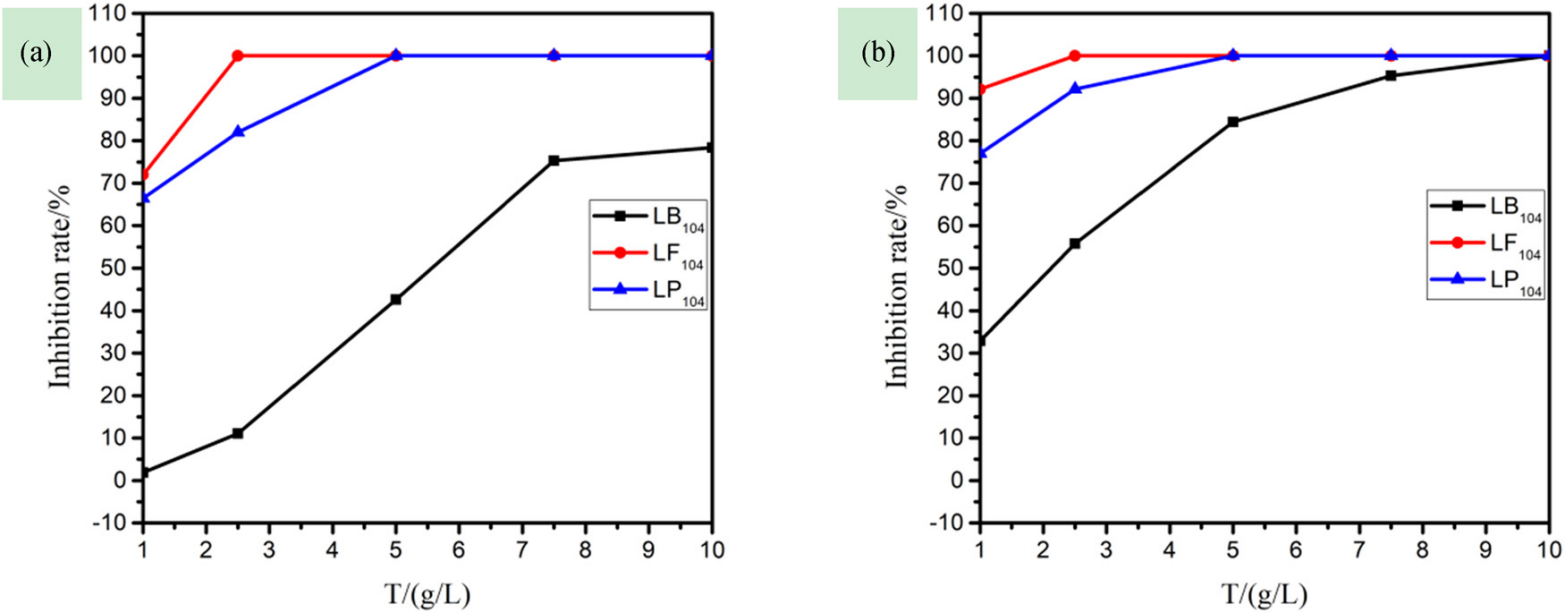
The relationship among the inhibition rate of rice stem length (a), the inhibition rate of rice root length (b), and the concentration of three kinds of ionic liquids.

The quality of the root is important for the normal growth and the development of the plant. Roots are also the main organs that are first subjected to pollutants [32]. Figure 3 shows that at a concentration of 1.00 g/L, only the inhibition rate of LB_104_ on plant stems was less than 50%, which has a smaller effect on the rice stem length; for IILs at the same concentration, their inhibition rate on the root system was higher than that on the stem length, that is, the damage of the ILs to the plant root system is greater [33]. Also, in terms of the sensitivity of rice stems and roots, the sequence of the three anions was as follows: [TF_2_N]- > [PF_6_]- > [BF_4_]-. Comparing with Figures 1 and 2, there are correlations: the germination rate and the root and stem lengths of the plant decrease sharply with the increase in the ILs concentration, and the root and stem lengths of the plant are negatively correlated with the inhibition rate.

#### 3.1.2 Toxic effects of three IILs on capsicum

(1) Effects of three IILs on germination rate and rooting rate of capsicum seeds

**Figure 4:**
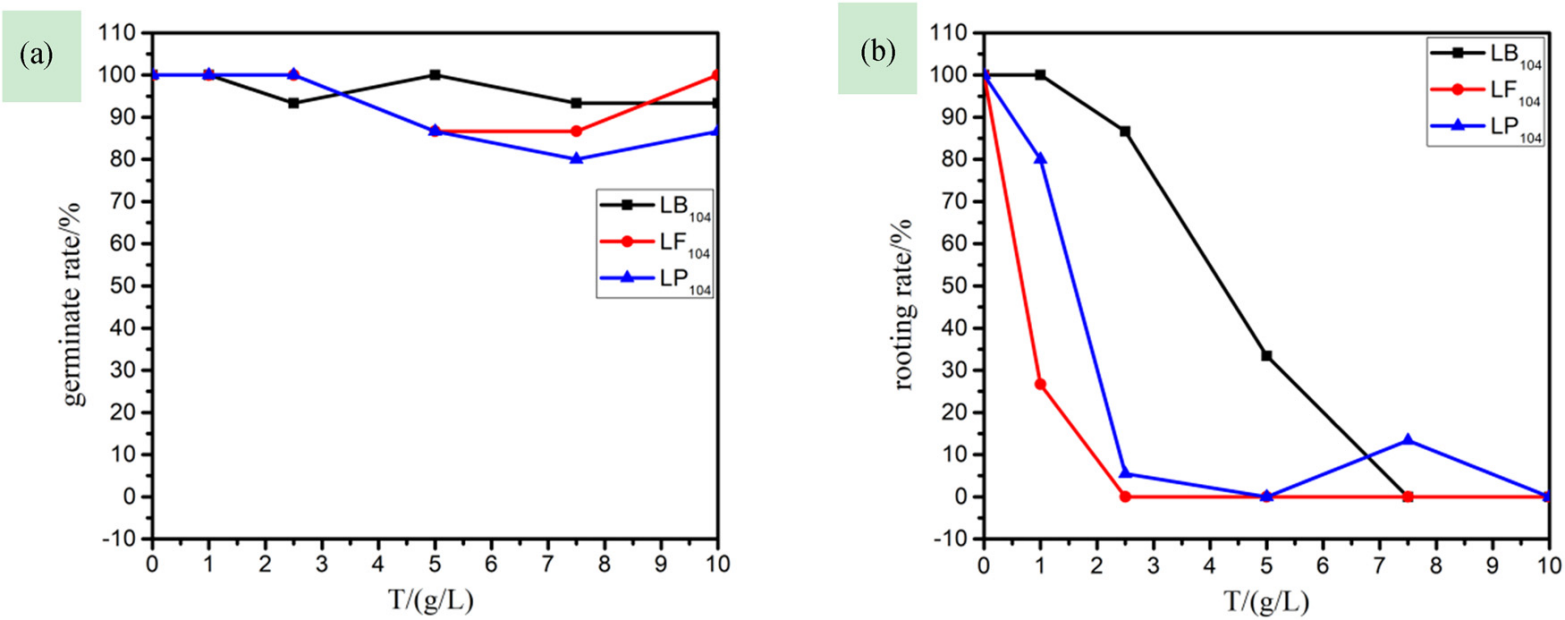
The relationship among germination rate (a), rooting rate (b), and the concentration of three kinds of ionic liquids in capsicum.

Figure 4 shows that the three IILs have a great effect on the root system of capsicum seeds. When the concentration of LP_104_ and LF_104_ solutions was 2.50 g/L, the capsicum seeds were completely rootless, but with little effect on the capsicum stem; also, in terms of the sensitivity to capsicum seeds, the sequence of the three anions was as follows: [TF_2_N]- > [PF_6_]- > [BF_4_]-. Matzke et al. [18] studied the toxic effect of different IIL anions on wheat and evaluated the differences in toxicity; the results showed that IM_14_(CF_3_SO_2_)_2_N was the most toxic, while IM_14_BF_4_ was mainly dominated by cations.

(2) Effects of three IILs on stem and root length of capsicum seedlings

**Figure 5:**
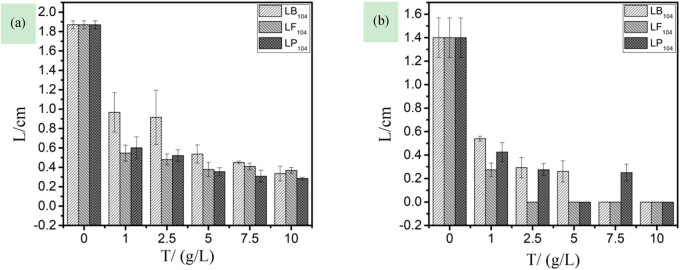
The relationship among stem length (a), root length (b), and the concentration of three kinds of ionic liquids in capsicum.

Figure 5 shows that with the same concentration in capsicums, their stem length was longer than the root; also, the plant stem and root lengths rapidly decreased with the increase in IIL concentration, and the IILs have a greater effect on the root system of rice [34]. With the increasing concentration, the capsicum roots become smaller, the lateral roots become fewer, and root deformities appear at the same time. LB_104_ had little effect on the two plants. Among the three IILs, LB_104_ has smaller effects on two plants than the other two. In terms of the sensitivity, the sequence of the three anions was as follows: [TF_2_N]- > [PF_6_]- > [BF_4_]-.

(3) Effects of three IILs on the inhibition rate of capsicum stems and roots

**Figure 6:**
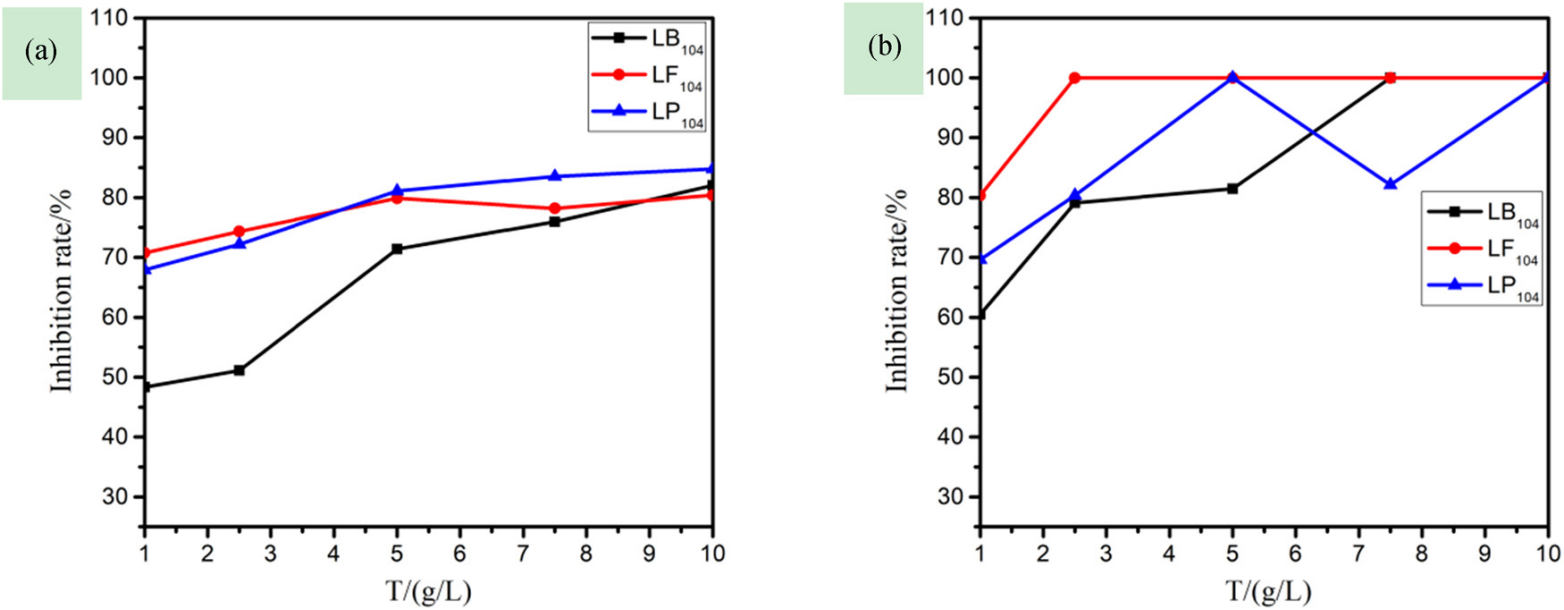
The relationship among the inhibition rate of capsicum stem length (a), the inhibition rate of capsicum root length (b), and the concentration of three kinds of ionic liquids.

Figure 6 shows that the inhibition rate of LB_104_ on capsicum was smaller than that of the other two; the ILs with the same concentration had a greater inhibiting effect on root system than that on the stem, that is, the damage of the ILs to the roots of the plant is greater, which is consistent with Figure 3. Besides, in terms of the sensitivity to stems and roots of the two plants, the sequence of the three anions was as follows: [TF_2_N]- > [PF_6_]- > [BF_4_]-.

### 3.2 EC_50_ concentrations of three IILs on two kinds of plants

The semi-inhibiting EC_50_ is the most common acute (short-term) toxicity test [30]. Both Tables 2 and 3 demonstrate that the sensitivity of the three ILs to the two plants varies. The sensitivity ranking of three IILs in rice was as follows: [TF_2_N]- > [PF_6_]- > [BF_4_]-, probably because the high chemical stability of sulfonate anion endowed by three IILs may lead to resistance to biotic or abiotic degradation and then increase its inherent toxicity. Both roots and stems conformed to this rule. When the concentration of LF_104_ was 1.038 g/L, half of the root and stem lengths of rice were inhibited. For capsicum, the sensitivity ranking of the three anions was [BF_4_]- > [TF_2_N]- > [PF_6_]-. When the concentrations of LB_104_ were 0.830 and 1.438 g/L, respectively, half of the capsicum roots and stems were inhibited. Comparing Tables 2 and 3, it is found that the three IILs had more severe inhibition of the root length than the stem length of rice and capsicum plants, and EC_50_ of the stem length was greater than that of the root length, indicating that the root system of rice seedlings is more toxic and sensitive than stem and leaves, and IILs have more toxic effects on plant roots than stems [35]. It may be because the root system of the plant is in direct contact with the IILs, while the plant mainly relies on the root system to absorb water from the soil and to provide physiological activities such as plant growth and metabolism and transpiration [36].

**Table 2:** EC_50_ of three IILs on stems of two kinds of plants

Ionic liquids	Monocotyledon	Dicotyledon
	Rice (EC_50_)	Capsicum (EC_50_)
LB_104_ (g/L)	4.986	1.438
LF_104_ (g/L)	1.038	2.540
LP_104_ (g/L)	2.450	3.465

**Table 3:** EC_50_ of three IILs on roots of two kinds of plants

Ionic liquids	Monocotyledon	Dicotyledon
	Rice (EC_50_)	Capsicum (EC_50_)
LB_104_ (g/L)	3.230	0.830
LF_104_ (g/L)	1.038	1.037
LP_104_ (g/L)	2.450	2.523

### 3.3 The toxicity of three IILs to the cell membrane of two kinds of plants

In the toxicity test of three IILs on rice, the three IILs have the greatest effect on the roots of rice and capsicum. This section studies the permeability of the root cells of rice and capsicum to the three IILs and further explores their toxicity.

**Figure 7:**
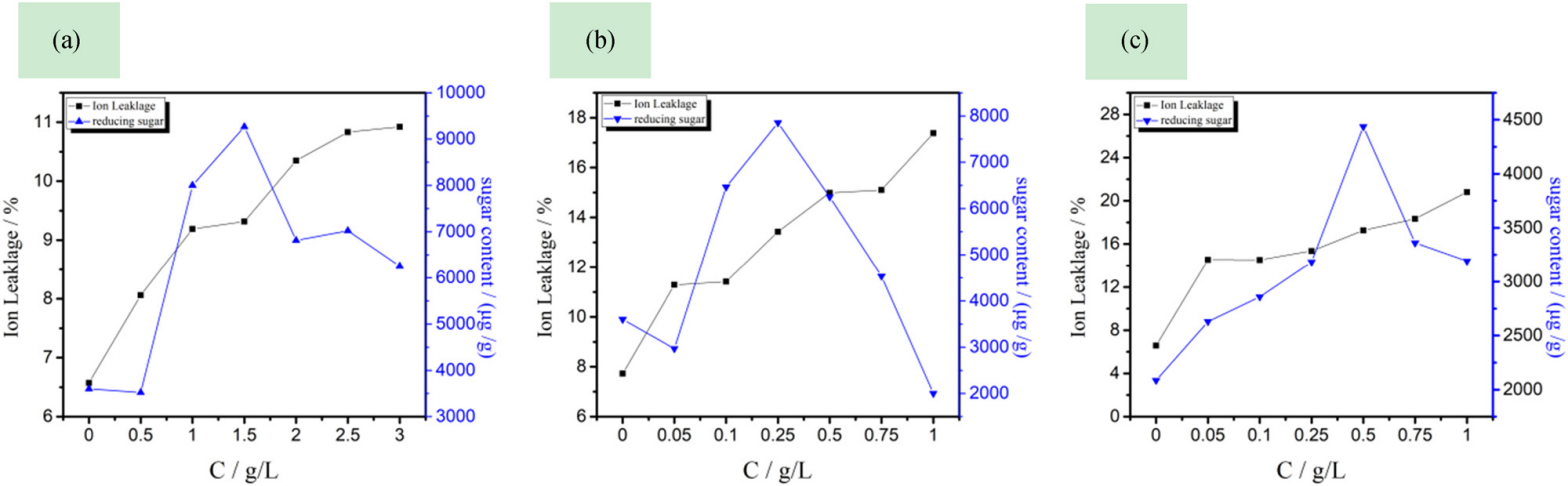
Effects of LB_104_ (a), LF_104_ (b), and LP_104_ (c) on membrane permeability of rice root cells.

Figure 7 shows that as the concentrations LB_104_, LF_104_, and LP_104_ increased, the electrical conductivity and the reducing sugar content in the bath solution of rice roots continued to increase; as their concentration was 1.038, 2.038, and 2.530 g/L, respectively, the reducing sugar content in the root bath solution reached a peak value, indicating that the IIL will destroy the permeability of the plant root cell membrane, and the cell membrane gradually breaks, that is, first, small molecules exudates, and as the concentration increases, the cell membrane damage increases, large molecular substances, for example, reducing sugars [37], begin to permeate, which in turn affects the plant roots. Electrolyte penetration in cells may be due to the ionic liquid being adsorbed on the surface of the lipid bilayer and interacting with membrane proteins, damaging the normal physiological functions of the cell membrane structure, causing the leakage of inorganic salts and reducing sugars, destroying rice root cells, and causing the irregular growth of plants until they withered and died [38].

**Figure 8:**
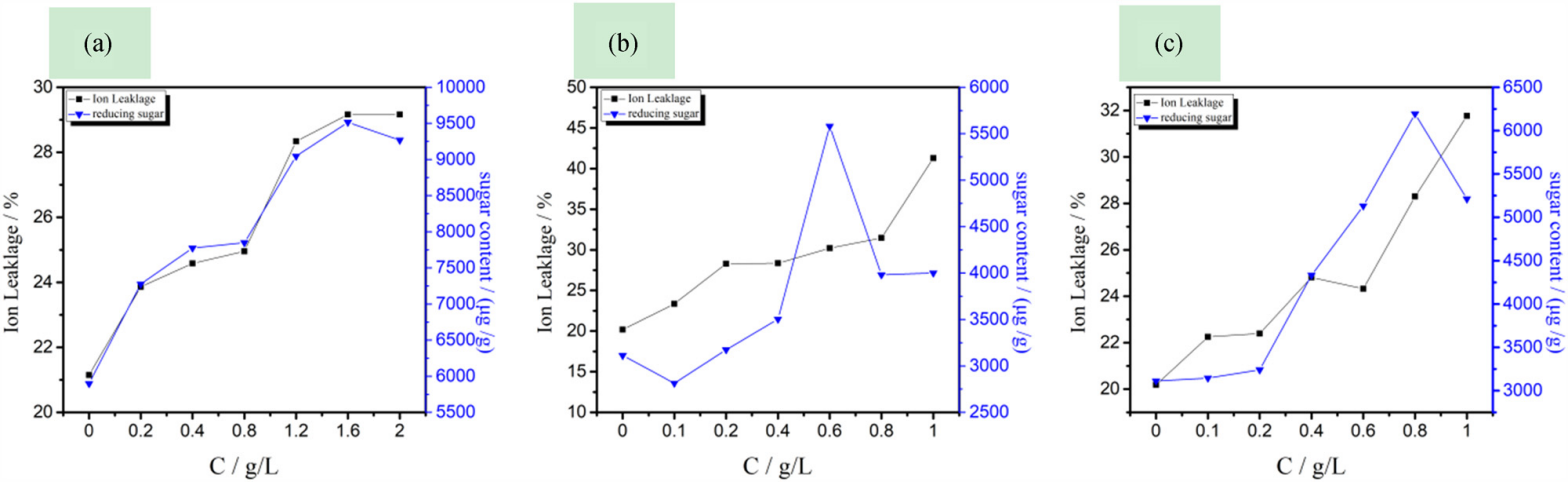
Effects of LB_104_ (a), LF_104_ (b), and LP_104_ (c) on membrane permeability of capsicum root cells.

As mentioned earlier, LB_104_, LP_104_, and LF_104_ have negative effects on the membrane permeability of capsicum plant cells. The influence of three kinds of IILs increases with the increase of IIL concentration on the membrane of capsicum root cells. Figure 8 shows that the more the concentration of IILs increases, the more electrolyte content in root cells is exuded. At the same time, the effect of the reducing sugar content showed the same trend. When the LB_104_ concentration was 0.720 g/L, the LF_104_ was 1.030 g/L, the LP_104_ reached 1.530 g/L, and the reducing sugar content increased to a peak, perhaps because the IIL destroys the cell membrane and expands its pores. Sugar is the metabolic center of organic substances in the plant body. It is not only the biosynthetic substance of phenolics, phytoalexin, lignin, and cellulose but also the carbon skeleton of proteins and nucleic acids. The permeation of carbohydrate destroys root cells and further affects the normal growth of plants. This conclusion is consistent with that in terms of the cell permeability of rice. Comparing Figure 7 with Figure 8, the homologous IL has a more serious effect on the permeability of the capsicum cell membrane.

## 4 Conclusions

The effects of three IIL anions on the germination and the seedling growth of rice and capsicum seeds were investigated. The three anions had obvious inhibitory effects on seed germination and growth. The higher the concentration of IIL, the more obvious the inhibition of germination rate, root length, stem length, root, and stem inhibition rate. By increasing the concentration of IILs, the conductivity of the bath solution of plant roots increased first, and then the content of the reducing sugar in root cells increased significantly with the destruction of the cell membrane.

Therefore, we conclude that the sensitivity sequence of the three anions to the two plants varies; in terms of the inhibiting effects of the three anions on rice, the sequence was [TF_2_N]- > [PF_6_]- > [BF_4_]-, while in terms of the capsicum, it was [BF_4_]- > [TF_2_N]- > [PF_6_]-. Comparing these two plants, capsicum is more sensitive to these three ionic liquids.
